# Plant Fungal Pathogenesis

**DOI:** 10.1155/2017/9724283

**Published:** 2017-01-17

**Authors:** Jun Yang, Tom Hsiang, Vijai Bhadauria, Xiao-Lin Chen, Guotian Li

**Affiliations:** ^1^Department of Plant Pathology, China Agricultural University, Beijing 100193, China; ^2^School of Environmental Sciences, University of Guelph, Guelph, ON, Canada N1G 2W1; ^3^Semiarid Prairie Agricultural Research Centre, Agriculture and Agri-Food Canada, Swift Current, SK, Canada S9H 3X2; ^4^College of Plant Science and Technology, Huazhong Agricultural University, Wuhan 430070, China; ^5^Department of Plant Pathology and the Genome Center, University of California, Davis, CA 95616, USA

Fungal plant pathogens can cause enormous losses in yield and quality of field crops, fruits, and other edible plant material, and this becomes increasingly a more important issue to human health and the global economy in this century, with increasing human populations and climate change threats to arable land. Deciphering fungal pathogenesis not only allows us to better understand how fungal pathogens infect host plants but also provides valuable information for the control of plant diseases, including new strategies to prevent, delay, or inhibit fungal development. This special issue summarizes recent novel findings on plant fungal pathogenesis.

Pathogenic fungi differ greatly in their life styles. Some are necrotrophic, while others are hemibiotrophic, biotrophic, or obligately biotrophic. Despite the obvious differences in life styles, fungal pathogens are known to use well-conserved proteins in infection processes. The conserved proteins are therefore potential targets for controlling these fungal diseases. X. Zhang et al. focused on receptor for activated C kinase 1 (Rack1), a conserved protein involved in various biological processes in eukaryotes. They reviewed functions of Rack1 proteins in model and pathogenic fungi. Rack1 proteins are involved in vegetative growth, conidiation, mating, toxin biosynthesis, and stress responses via different pathways including cAMP/PKA and MAPK pathways in different fungi, illustrating how Rack1 proteins are involved in fungal pathogenesis.

In order to infect, pathogenic fungi can develop specialized infection structures, such as appressoria to penetrate host cells. During this process, the peroxisomes play key roles to facilitate full functions of virulence proteins. X.-L. Chen et al. focused on roles of the peroxisomes in the rice blast fungus* Magnaporthe oryzae*. They described molecular mechanisms underlying how the peroxisomes function related to life cycles and metabolic processes. And also, they provided an overview of the relationship between peroxisomes and pathogenicity. This review will be valuable for researchers interested in understanding how the peroxisomes serve as a platform to orchestrate plant host invasion by plant filamentous fungi.

The hemibiotrophic fungus* Colletotrichum higginsianum* is the causal agent of anthracnose diseases on a wide range of cruciferous plants (Brassicaceae), including the model plant* Arabidopsis thaliana. *Also, the* C. higginsianum*-*A. thaliana* pathosystem is now considered to be an important model for studying fungal pathogenicity, in which both hosts can be efficiently genetically manipulated. The conserved pathogenic factors in various fungal pathogens that have been the subject of much study might also be considered targets for pesticide design. As a pleiotropic regulator of morphogenesis and plant infection, Ste7 MEK possesses highly conserved roles in phytopathogens. Q. Yuan et al. reported that the* C. higginsianum *gene* ChSTE7* is involved in regulation of vegetative growth, appressorial formation, and invasive growth in host tissues. This is an important and conserved virulence factor affecting infection of* C. higginsianum *on cruciferous plants.

Covalent histone modifications, such as methylation and acetylation, provide key epigenetic information in transcriptional regulation and chromatin structure organization for functional responses. Histone methylation provides an excellent epigenetic mechanism for stable transfer of gene expression profiles to progeny cells. In pathogenic fungi, the SET domain-containing proteins play essential roles in fungal growth and development. Z. Cao et al. demonstrated that MoKMT2H, an Ash1-like histone modification protein, played important roles in conidial germination and pathogenesis in* M. oryzae*. They found that the Δ*Mokmt2h* null mutants are not defective in genome-wide histone methyltransferase modification, vegetative hyphal growth, conidial morphology, conidiation, or disease lesion formation on rice leaves. However, the* MoKMT2H *deletion mutants showed delayed conidial germination and attenuated virulence. Their results suggested that* MoKMT2H* plays an important role in conidial germination in the rice blast fungus.

The defense mechanisms of wheat against* Puccinia striiformis *f. sp.* tritici* (*Pst*) infection are complex, and activation of defense responses is critical in order to prevent the spread of pathogens. The plant cytoskeleton, including microtubules and microfilaments, is a highly dynamic subcellular structure that is associated with plant defense responses. J. Wang et al. focused on the function of microtubule polymerization in wheat against the stripe rust fungus* Pst* CYR23. They detected the frequency of hypersensitive cell deaths and H_2_O_2_ accumulations in leaves treated with microtubule inhibitor oryzalin before inoculation with strain CYR23. Depolymerization of microtubules reduced the resistance of plants via hypersensitive responses and led to decreased H_2_O_2_ accumulation, suggesting that microtubules play roles in resistance against the stripe rust fungus in wheat.

Taken together, these research results have furthered our understanding of plant-microbe interactions and may help promote further research in this area.

*Jun Yang* *Tom Hsiang* *Vijai Bhadauria* *Xiao-Lin Chen* *Guotian Li*

